# Research on Tool Wear Monitoring Technology Based on Variational Mode Decomposition and Back Propagation Neural Network Model

**DOI:** 10.3390/s24248107

**Published:** 2024-12-19

**Authors:** Kang Wang, Aimin Wang, Long Wu

**Affiliations:** 1 Digital Manufacturing Institute, Beijing Institute of Technology, Beijing 100081, China; wangkang933@126.com; 2 School of Mechanical and Electrical Engineering, Shandong Jianzhu University, Jinan 250101, China; wulong0128@163.com

**Keywords:** tool wear prediction, VMD-BP neural network, spindle power, spindle current, dynamic monitoring

## Abstract

Accurately predicting tool wear during the machining process not only saves machining time and improves efficiency but also ensures the production of good-quality parts and automation. This paper proposes a combined variational mode decomposition (VMD) and back propagation (BP) neural network model (VMD-BP), which maps spindle power to tool wear. The model is trained using both historical and real-time data. To improve accuracy, the internal power data from the machine tool are used to calibrate the model’s input data. Data collected from milling experiments are used to test the model, with sensor-collected power being compared to the model’s predicted power. The average error was 1.1256%, which confirms the reliability of the model. In practical applications, the model enables the real-time monitoring of spindle power, helping prevent excessive tool wear during machining. This offers significant guidance for actual production processes.

## 1. Introduction

In the manufacturing industry, CNC milling is widely applied, with the cutting tool being an indispensable part of the production process. Real-time monitoring of the tool wear status is particularly important for production machining. During the machining process, downtime caused by tool failure accounts for approximately 7–20% of the total time taken [1], which not only wastes machining time but can also, in severe cases, damage the workpiece or the machine tool. Therefore, the implementation of tool wear monitoring during the machining process is of significant importance.

According to different measurement methods, tool wear monitoring systems (TWMSs) can be divided into “direct measurement methods” and “indirect measurement methods”. Due to the numerous inconveniences and time consumption associated with “direct measurement methods”, most scholars have conducted more research on “indirect measurement methods”. During the machining process, a variety of parameters can be measured to reflect the tool’s condition, such as acoustic emission (AE), cutting force, vibration signals, sensor fusion techniques, thermal imaging, spindle torque/current, power, etc. [2].

Prasad et al. [3] utilized acousto-optic emission sensors (i.e., laser Doppler vibrometers) for online tool condition monitoring, encompassing data acquisition and signal processing. They analyzed the surface topography during machining by integrating surface metrology with online metrology tools, enabling the comprehensive measurement of various aspects of the machining process. Zhang et al. [4] proposed a method to detect a tool’s life stages using only cutting force data, determining the usability of undamaged micro-milling tools. By feeding cutting force data into machine learning models, they classified tool conditions into different stages, achieving online monitoring of tool conditions during micro-milling. Mohanraj et al. [5] extracted features such as wavelet coefficients, Hölder exponents, and statistical characteristics from vibration signals for various machine learning algorithms, including an SVM, a KNN, kernel-based Bayesian methods, a multilayer perceptron, and decision trees, to predict tool wear. They compared the accuracies of these algorithms. Cao et al. [6] introduced an intelligent technique to identify tool wear conditions using spindle vibration signals. The proposed approach combined derived wavelet frameworks (DWFs) with convolutional neural networks (CNNs). The experimental results for tool wear state identification demonstrated the feasibility and effectiveness of this method. Xin et al. [7] presented a method for the sparse decomposition of vibration signals using a dual-basis pursuit algorithm and morphological component analysis. This approach utilized vibration signal features to monitor tool wear conditions. Liu et al. [8] developed a multi-sensor data fusion system for the online prediction of the remaining useful life (RUL) of machine tools. The reliability of the proposed system’s prediction performance was validated through case studies. Shankar et al. [9] designed an efficient tool wear monitoring system for the keyway milling of 7075-T6 hybrid aluminum alloy composites. By measuring sound pressure and cutting force during machining and employing sensor fusion technology, the system utilized MATLAB-based neural networks (MATLAB2020b) and ANFIS models to predict tool conditions. Ying et al. [10,11] analyzed the correlation between various signal features and tool wear, confirming the feasibility and effectiveness of spindle power as one indicator for evaluating tool conditions. Additionally, they established an energy consumption model considering tool wear, where spindle power served as the sole indicator for identifying tool wear states. Genetic algorithms were employed to optimize machining parameters and strategies for different wear stages.

Drouillet et al. [12] utilized spindle power sensor data combined with artificial neural network (ANN) curve-fitting methods to predict the remaining useful life (RUL) of tools during milling operations. Corne et al. [13] conducted real-time predictions of tool wear/breakage during the drilling of Inconel superalloys by collecting spindle power data, which they compared with force data to validate the reliability of spindle power signals. Yuan et al. [14] proposed a tool wear monitoring method that integrates variational mode decomposition (VMD) with ensemble learning. This method extracted tool condition features from spindle motor current signals and established a nonlinear prediction model, demonstrating superior accuracy and robustness under machining conditions compared to traditional methods. Shao et al. [15] developed a cutting power model for tool wear monitoring under varying cutting conditions. They simulated intermittent cutting loads on the spindle motor during milling, showing through simulation that average cutting power signals provide better tool wear predictions than instantaneous cutting power signals. The model was successfully implemented under different cutting conditions. Dehua et al. [16] introduced a Gaussian process regression model-based method for predicting the RUL of tools under varying cutting conditions. By integrating the tool wear mechanism and assuming a progressive tool wear process, the covariance matrix of the Gaussian model constrained predictions at adjacent time points to linear relationships, effectively simulating wear processes under continuous variation. Chang Hao et al. [17] addressed the non-stationary nature of milling vibration signals by optimizing VMD parameters using differential evolution algorithms. Coupled with a naive Bayes classifier, the method achieved high-accuracy wear state recognition, with significantly enhanced denoising capability and feature extraction. Shui Xing et al. [18] optimized VMD parameters with an improved whale optimization algorithm, extracted features using multiscale permutation entropy, and achieved a 98.44% recognition rate for wear states through one-dimensional convolutional neural networks, outperforming traditional methods. Bazi et al. [19] developed a hybrid deep learning model combining a CNN and BiLSTM with VMD. By synergizing signal decomposition and temporal modeling, this approach significantly improved tool wear prediction performance and generalization capabilities. Cai Lihong et al. [20] adopted the sparrow search algorithm to optimize VMD parameters and efficiently extracted and classified tool vibration signal features using support vector machines, achieving a 93.3% recognition accuracy, demonstrating strong practical value. Data processing is also very important. Yang et al. [21] proposed an improved Generative Adversarial Network (IGAN) combined with an Enhanced Deep Extreme Learning Machine (EDELM) to address the issue of data imbalance in fault diagnosis. Their method successfully rebalanced the dataset, resulting in improved accuracy. Zhang et al. [22] developed a global modeling strategy that integrates Dynamic Kernel Canonical Variate Analysis (GDKCVA) for fault detection. This approach considers both time-wise and batch-wise dynamics, effectively addressing the nonlinear relationships between time dynamics and variables. Zhang et al. [23] introduced an improved Extreme Learning Machine (HRIELM) based on hybrid resampling for fault diagnosis under imbalanced data conditions. By combining a novel resampling technique with ensemble learning, their method improved diagnostic accuracy and highlighted the importance of balanced data for reliable fault detection in cooling systems.

**Figure 1:**
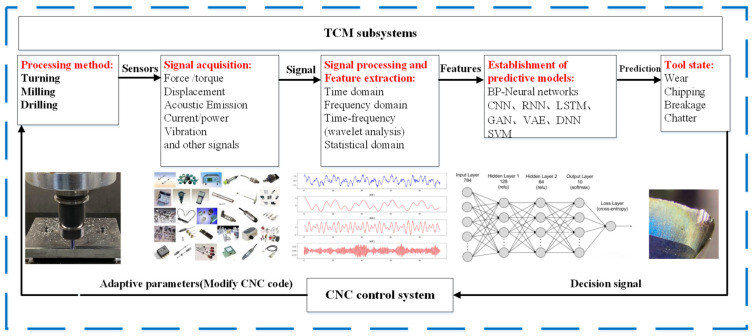
Overview of a TWMS for machining operation.

In summary, as illustrated in Figure 1, a typical TWMS consists of signal/data acquisition, signal processing, feature extraction, and decision-making or classification components. The data or signal acquisition process is hardware-based, while the remaining TCMS components are software-driven.

However, the aforementioned studies have several limitations, which can be summarized as follows: (1) Regarding signal or data acquisition, measurement sensors such as optical microscopes, CCD cameras, force sensors, vibration sensors, acoustic emission sensors, and power sensors are utilized. However, these sensors are not sufficiently practical for industrial applications. (2) In indirect measurement methods, force sensors have been well tested and are considered the most accurate. However, their application is primarily confined to research laboratories. The requirements for multiple operations and downtime for force sensors pose a major limitation to their industrial usage. (3) Power or current measurement methods are simpler. For TWMSs, these methods are relatively easier to develop, cost-effective, and reliable and so have attracted significant attention from researchers. Preliminary results are comparable to those obtained using force data. However, further studies in industrial applications are required [24]. (4) Most of the aforementioned studies require modifications to the machine tools, which are unsuitable for practical machining. In practice, excessive modifications to operating machine tools are not recommended, as they can limit the widespread adoption of these methods.

To address these limitations, this study proposes a VMD-BP neural network model. This model utilizes a Hall sensor to collect spindle current data for calculating spindle power and establishes a mapping relationship between spindle power and tool wear to predict tool wear [25]. This method does not require modifications to the machine tool, making it highly versatile. By combining the VMD signal processing method with the BP neural network model and using the corrected spindle current data to predict tool wear, the complexity of the tool wear prediction model is significantly simplified. When used for prediction, the model’s computation time is approximately 0.15 s, achieving near-real-time performance while still maintaining high accuracy.

The structure of this paper is as follows: Section 2 introduces the model development process. Section 3 focuses on the collection and organization of experimental data. Section 4 presents the testing of the model and analyzes the mapping relationship between tool wear and spindle power. The experimental validation demonstrates the accuracy of the proposed model using power data collected from CNC milling machines.

## 2. Development of the VMD-BP Neural Network Model

### 2.1. Introduction to the VMD Algorithm

Variational Mode Decomposition (VMD) is an advanced signal processing technique that addresses some limitations of traditional methods such as Empirical Mode Decomposition (EMD). VMD is an adaptive signal decomposition approach that breaks down complex signals into a set of Intrinsic Mode Functions (IMFs), which represent different frequency components of the original signal. Unlike EMD, VMD avoids mode mixing issues and is capable of handling non-stationary and nonlinear signals, making it particularly suitable for processing complex signals encountered in mechanical systems, such as power and current signals collected during milling operations.

The core idea behind VMD is to decompose the original signal into multiple IMFs, each characterized by a central frequency and bandwidth. VMD employs variational methods in the frequency domain to decompose the signal, minimizing the bandwidth of each mode. The optimization objective of VMD can be expressed as [18]

(1) $$\underset{\left\{u_{k}\},\{\omega_{k}\right\}}{\min}\sum_{k-1}^{K}{\left\|x(t)-\sum_{k-1}^{K}u_{k}(t)\right\|}^{2}+\lambda \sum_{k=1}^{K}{\left\|\frac{\partial }{\partial t}u_{k}(t)\right\|}^{2}+\alpha \sum_{k-1}^{K}{\left(\omega_{k}-{\hat{\omega }}_{k}\right)}^{2}$$

where 

$u_{k}(t)$ represents the k−th Intrinsic Mode Function (IMF);

$\omega_{k}$ is the center frequency of the k−th mode;

${\hat{\omega }}_{k}$ denotes the estimated central frequency for each mode;

α is a regularization parameter controlling the smoothness of each mode;

λ regulates the bandwidth of each mode. The first term in the objective function minimizes the reconstruction error between the original signal, x(t), and the sum of all IMFs. The second term penalizes high-frequency fluctuations within each mode, promoting smoothness. The third term ensures that the central frequency of each mode remains close to its specific value.

The decomposition process in VMD iteratively adjusts the IMFs, $u_{k}(t)$, and their corresponding frequencies, $\omega_{k}$, through an alternating minimization method to minimize the cost function. This process includes the following steps:

Step 1: Initialization: initialize the IMFs, $u_{k}(t)$, and their corresponding center frequencies, $\omega_{k}$.

Step 2: Frequency Update: update the center frequency, $\omega_{k}$, of each mode by minimizing the frequency error.

Step 3: Mode Update: update each mode, $u_{k}(t)$, by minimizing the reconstruction error and ensuring smoothness.

Step 4: Convergence: iterate through the above steps until the decomposition converges, meaning the IMFs no longer exhibit significant changes.

Ultimately, VMD yields a set of sub-signals, $u_{k}(t)$, each corresponding to a specific frequency band of the original signal. These IMFs can then be analyzed individually to extract feature values, which serve as input features for subsequent neural network learning models.

### 2.2. Training the VMD-BP Neural Network Model

Tool life refers to the amount of time taken for the flank wear land to reach a predefined width. Hence, the prediction of tool condition and tool life is closely related to identifying the tool wear land [26]. During machining, the evolution of typical tool wear consists of three stages: the initial wear stage, the steady wear stage, and the accelerated wear stage [27]. Researchers have developed various tool wear modeling methods, such as empirical and physical wear models. These methods rely on practical experience and technical measurements to generalize tool wear [28].

Improving the accuracy of tool wear monitoring critically depends on the selection of an appropriate tool wear model. Developing a tool wear model is a complex process requiring extensive cutting experiments to establish the relationships between variables, parameters, and the mathematical equations of the wear model.

**Figure 2:**
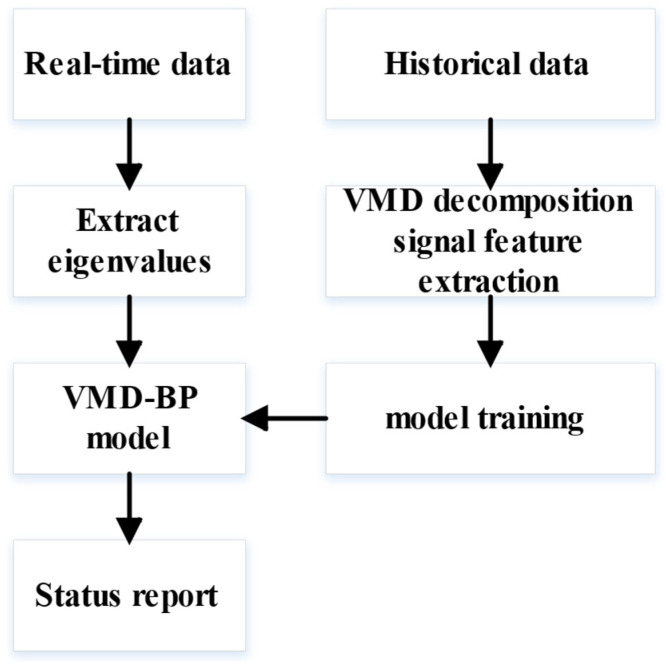
Training process of the VMD-BP neural network model.

A typical neural network structure consists of an input layer, hidden layers, and an output layer. The training process for constructing a multivariate linear regression model based on a neural network is illustrated in Figure 2. The proposed model is trained using historical measured data, wherein the data are decomposed and feature values are extracted using VMD. These features are then used to construct the VMD-BP neural network model.

During real-time operation, the model extracts features from live data collected during the machining process and inputs them into the pre-trained neural network model for wear prediction. This approach ensures accurate and efficient tool wear monitoring and prediction.

The BP neural network model is characterized by its simple structure and fast computational speed, making it suitable for real-time data processing. The model construction formula is as follows:

(2) $$y=W\cdot X+b=w_{1}x_{1}+w_{2}x_{2}+w_{3}x_{3}+w_{4}x_{4}+w_{5}x_{5}+b$$

where W represents the weight between neurons; b is the bias associated with a neuron; X is the input value; and y is the output of the neural network.

This formula illustrates how the input data, X, are processed through the network, with weights and biases adjusted during the training process to optimize the output, y, for accurate tool wear prediction.

**Figure 3:**
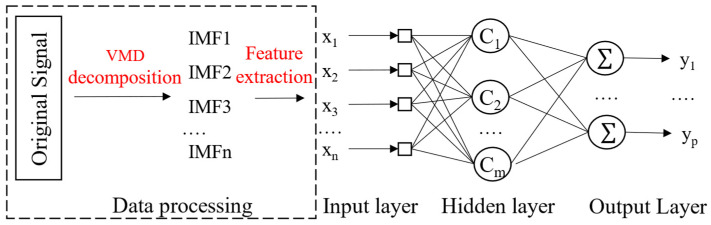
Basic structure of the VMD-BP neural network model.

As shown on the right side of Figure 3, $X_{n}$ represents the input layer data, $C_{m}$ is the hidden layer, and $Y_{p}$ is the output layer in the feedforward neural network. The following parameters are defined:

The weight between the input layer and the hidden layer is denoted as W;

The bias term for the hidden layer is $b_{1}$;

The activation function for the hidden layer is $g_{1}$;

The weight between the hidden layer and the output layer is V;

The bias term for the output layer is $b_{2}$;

The activation function for the output layer is $g_{2}$.

Thus, from the input layer to the hidden layer, the relationship can be expressed as follows:

(3) $$net_{1}=W^{T}X_{n}+b_{1},h=g_{1}(net_{1})$$

and from the hidden layer to the output layer:

(4) $$net_{2}=V^{T}h+b_{2},Y_{p}=g_{2}(net_{2})$$

Therefore, the model in Figure 3 can be represented as follows:

(5) $$Y_{p}=g_{2}(V^{T}g_{1}(W^{T}X_{n}+b_{1})+b_{2})$$

In this model, we select the ReLU (Rectified Linear Unit) function as the activation function because of its sparsity, which reduces the interdependence of parameters, thus mitigating the overfitting problem. Additionally, it has a low computational cost and fast processing speed. The ReLU activation function is expressed as follows:

(6) $$g(x)=\left\{\begin{array}{l} x,x\geq 0\\ 0,x<0 \end{array}\right\}$$

Finally, the loss function used to train the model is given by

(7) $$L=\sum_{i=1}^{n}{\left(y-y_{\_}pre\right)}^{2}$$

where y is the true output value, $y_{\_}pre$ is the predicted output value, and n is the number of data points. This loss function is used to minimize the prediction error during model training.

The model is built in four steps, as shown below:

Step 1: Prepare the dataset: Classify and filter the historical data, use VMD to decompose the data and extract feature values, and organize the data according to the input format of the model in preparation for training.

**Figure 4:**
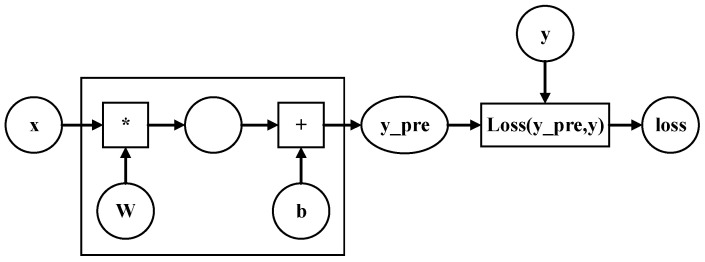
Solving the loss function.

Step 2: calculate the predicted value, $y_{-}pre$, and solve for the loss value, as shown in Figure 4.

Step 3: Back propagation algorithm solution to calculate the gradient of W and b: use the backpropagation algorithm to calculate the speed at which the loss function changes when the weights and biases are changed.

The partial derivatives of the loss function can be solved by

(8) $$\frac{\partial loss}{\partial b_{j}^{l}}=\delta_{j}^{l}$$

(9) $$\frac{\partial loss}{\partial w_{jk}^{l}}=\alpha_{k}^{l-1}\delta_{j}^{l}$$

where $b_{j}^{l}$ denotes the bias on the *j*-th neuron in layer *l*, $w_{jk}^{l}$ denotes the weight between the *k*-th neuron in layer (*l* − 1) and the *j*-th neuron in layer *l*, $\delta_{j}^{l}$ denotes the error value on the *j*-th neuron in layer *l*, and $\alpha_{k}^{l-1}$ denotes the activation value of the *k*-th neuron in layer (*l* − 1).

Step 4: Clear the gradient value, update the weights and biases, and enter the loop again. The weights and biases are updated by

(10) $$w_{jk}^{l}=w_{jk}^{l}-\alpha \frac{\partial loss}{\partial w_{jk}^{l}}$$

(11) $$b_{j}^{l}=b_{j}^{l}-\alpha \frac{\partial loss}{\partial b_{j}^{l}}$$

where α is the learning rate, which is used to regulate the intensity of the changes and avoid overshooting.

The process continues until the loss function is smaller than a predetermined threshold or the number of iterations is exhausted. The parameters at this point are considered the best parameters. After the loop ends, the average loss is output.

## 3. Experimental Research

### 3.1. Collection of Machining Experimental Data

**Figure 5:**
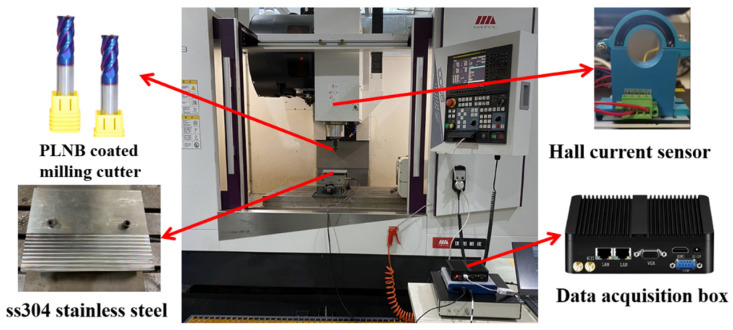
Machining experimental environment.

The machining experimental environment is shown in Figure 5. The material used is SS304 stainless steel, with dimensions of 200 mm in length, 140 mm in width, and 25 mm in thickness. The machine tool is a three-axis CNC machine (model: VMC10008), and the tool used is a φ8 four-flute nano-blue-coated end mill (PLNB), with a corner radius of R = 0.5 mm and a hardness of HRC 65. A Hall current sensor is used to measure spindle current, and a data acquisition box is used to collect sensor information from the machine tool for calibration. The machining parameters are selected based on commonly used milling parameters with variations, and a full factorial experiment is designed for tool testing. The machining parameters are listed in Table 1, and the experiment is designed as a three-factor, three-level test (where the cutting width remains constant and is not considered as a factor).

**Figure 6:**
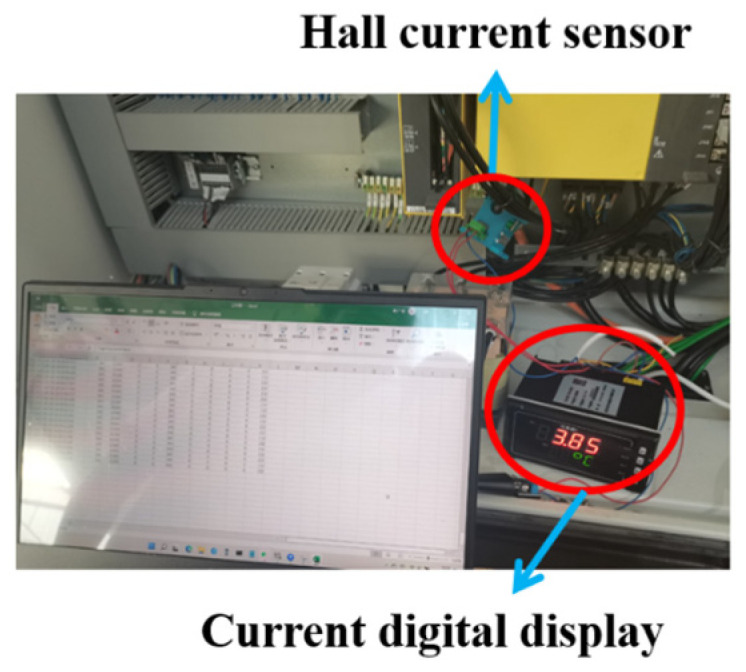
Spindle current acquisition system.

The Hall current sensor is used to measure the variation in spindle current during the machining process, as shown in Figure 6. The model of the current sensor is MIK-HRI-50A, with an output range of DC 4–20 mA. It is used in conjunction with a digital display and can be directly connected to a computer via RS485 communication. According to the machine tool parameter manual, the machine tool voltage is 380 V, and the power factor is set to 0.8. The spindle power of the machine tool can be calculated as follows [29,30]:

(12) $$P=\sqrt{3}UI\cos\theta$$

where U is the effective voltage; I is the effective current; and cosθ is the power factor.

**Figure 7:**
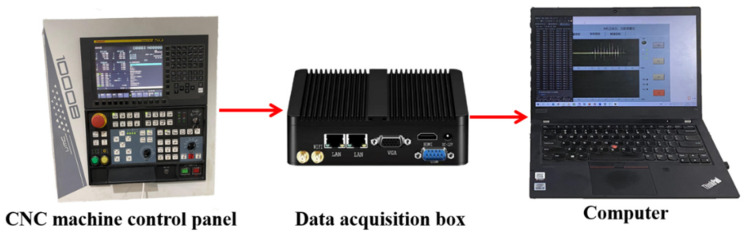
Machine internal information acquisition system.

The machine data acquisition box, as shown in Figure 7, is connected to the machine tool through a network cable to collect data during processing, including the position of the machine tool, the execution of NC code, and the load of the spindle and the three axes of xyz. The collected spindle load is used to correct the data from the Hall current sensor to ensure the accuracy of the collected current data.

**Figure 8:**
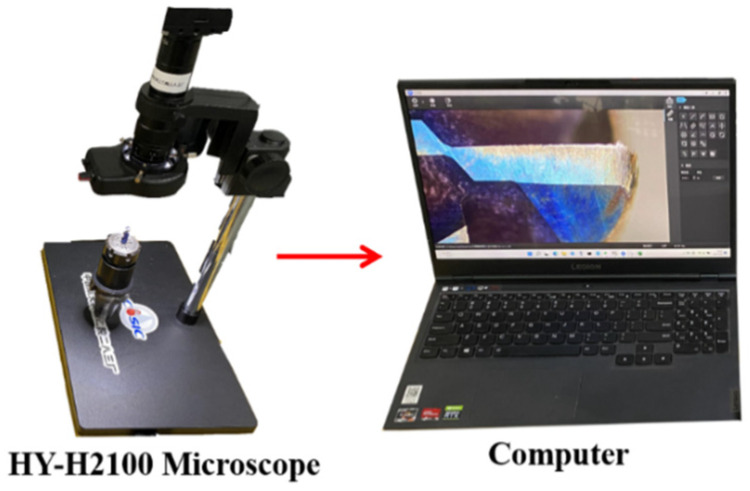
HY-H2100 electron microscope.

As shown in Figure 8, an HY-H2100 portable microscope for rear-tool-face wear measurement is used, and the tool wear measurement can be performed while cutting, improving the efficiency of the experiment.

The wear standard is determined based on the width of the flank wear land (VB). The width of the flank wear land is generally selected as 0.15 mm–0.2 mm as the wear limit. Therefore, if the measured value exceeds 0.2 mm, it indicates that the tool is approaching failure. This standard is used to arrange the milling experiments for the tool. The machining process parameters are fixed, and the workpieces are repeatedly milled. When the measured wear exceeds 0.2 mm and reaches 0.3 mm, the experiment is stopped, and the experimental data are organized and saved.

### 3.2. Data Processing of the Machining Experiment

**Figure 9:**
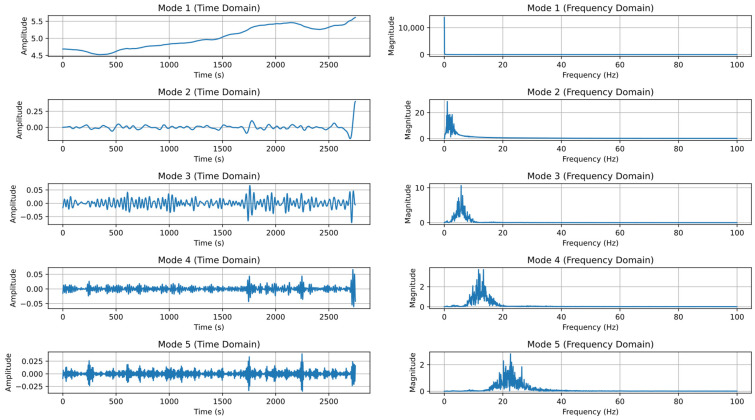
VMD-decomposed data.

Since the machining process is stable, the collected data (such as tool wear land width, VB, and spindle current, I) are classified and filtered. VMD is used to decompose the data and extract feature values, as shown in Figure 9, in preparation for subsequent model use.

**Figure 10:**
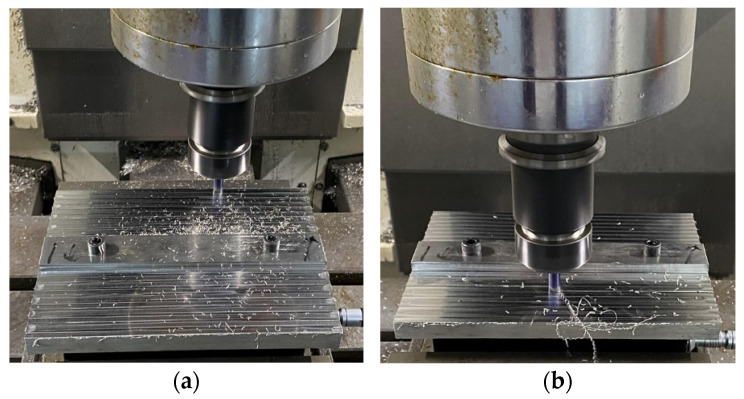

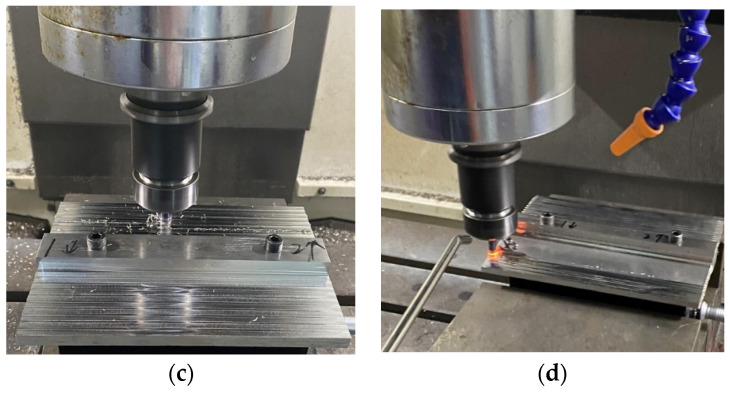
Changes in cutting behavior at different stages of tool wear: (**a**) tool at the initial machining stage; (**b**) tool at the later stages of normal wear; (**c**) tool during the rapid wear phase; (**d**) tool approaching failure.

**Figure 11:**
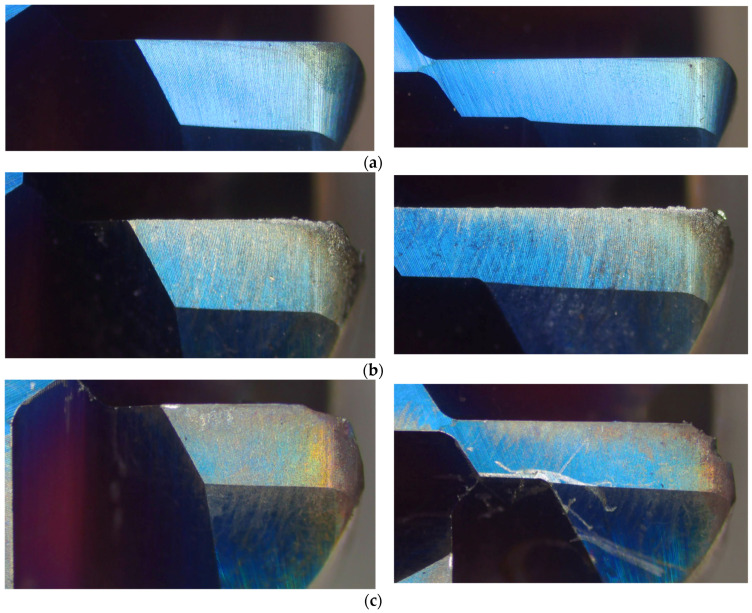

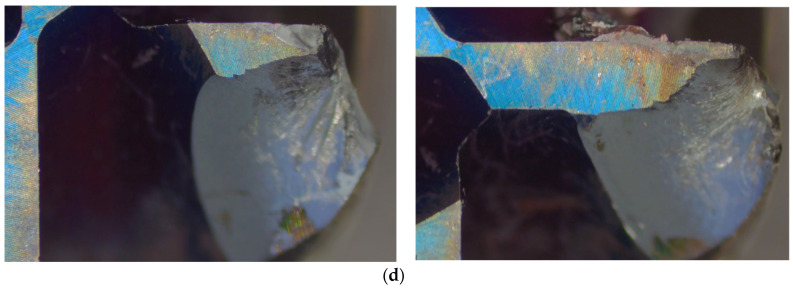
Tool wear conditions: (**a**) brand-new tool; (**b**) tool in normal wear condition; (**c**) rapid tool wear; (**d**) tool breakage.

It can be observed that when the tool is in brand-new condition or at the early stages of normal wear, the tool is relatively sharp and the chips are small, as shown in Figure 10a, where each chip exists separately. When the tool enters the later stages of the normal wear phase, the tool tip starts to develop notches and even chipping, as shown in Figure 11b. The resulting chips, as shown in Figure 10b, begin to form string-like shapes and no longer break into separate pieces. During the rapid wear stage, the chips, once formed, easily wrap around the tool and do not break apart, as shown in Figure 10c. Finally, when the tool is about to fail, the cutting force gradually increases, the tool wear increases, and the friction with the workpiece also increases, causing the tool to turn red, as shown in Figure 10d.

Here, data from one of the tools are used as a representative example. The tool wear conditions are shown in Figure 11. Subfigure (a) shows a photo of the new tool; (b) shows a photo of the tool when it enters the normal wear state; (c) shows the tool in the rapid wear state, approaching the critical wear point where the tool tip has completely broken off; and (d) is a photo of the tool in its final wear failure state, where the entire tool has fractured.

For each tool during the machining process, spindle current data are recorded during milling operations. These current data are used to calculate spindle power and validated using data collected by the acquisition device. In spindle power calculation, energy consumption models that consider tool wear factors primarily comprise two types: the basic exponential model [28] and the quadratic response surface model [29]. The basic exponential energy consumption model is the most commonly used due to its simplicity; it requires fewer coefficients and is easier to construct while being applicable to most CNC machine operating conditions.

Therefore, this paper adopts the basic exponential energy consumption model to establish a mapping relationship between spindle power and tool wear. Using the cutting power calculation Formula (13), cutting power can be solved based on machining parameters and tool wear values:

(13) $$P=K{\left(1+VB\right)}^{u1}a_{p}^{u2}a_{e}^{u3}v_{f}^{u4}v_{c}^{u5}$$

By inputting the machining parameters and measured flank wear width of each tool into the system for fitting and solving, the coefficients, u1,u2,u3,u4,u5, are determined, thereby establishing the mapping relationship between tool wear and spindle power.

To verify the feasibility of the experiment and demonstrate the tool wear condition, spindle power data were used for analysis. The experimental setup is as follows:

(1) For each tool, the machining parameters were fixed, and the material being cut was consistent.

(2) A fixed cutting length was processed each time, after which the flank wear width of the tool was measured and recorded.

(3) Simultaneously, spindle current data were saved in real time and used to calculate spindle power.

## 4. Results and Discussion

**Figure 12:**
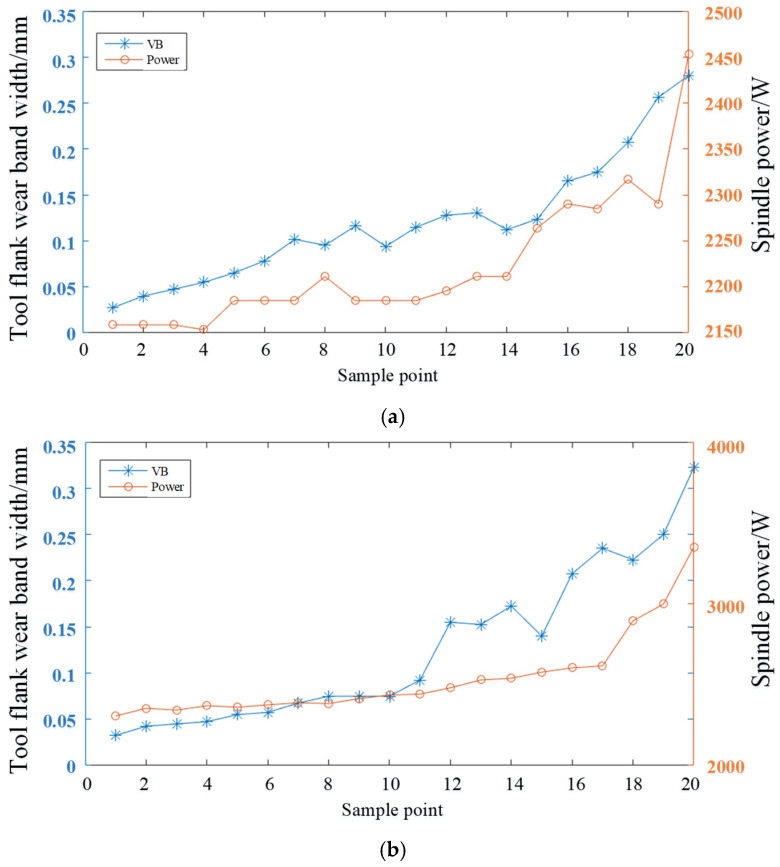
Wear and power variation trends for two sets of tools: (**a**) Set one; (**b**) Set two.

Based on the previous discussion, the correlation between spindle power and tool wear value was analyzed and verified. The complete dataset of the tools was examined, and the wear and power changes in two sets of tools are illustrated as examples in Figure 12.

The results indicate that as tool wear increases, the spindle power during machining also increases, showing a positive correlation between the two. This relationship suggests that the changes in spindle power during machining can be utilized to predict the degree of tool wear. By mapping spindle power to the flank wear width, the wear state of the tool can be effectively estimated.

**Figure 13:**
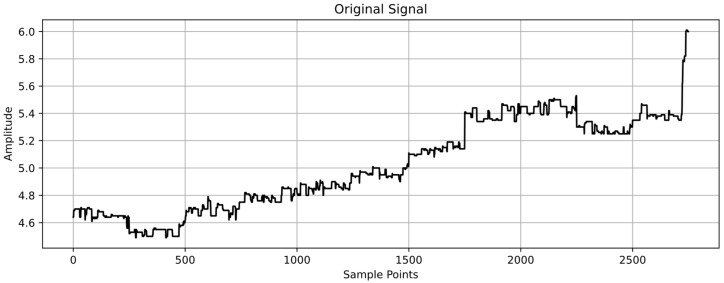
Spindle Current Variation Curve.

Data collected from the experiments designed in Section 3.1 were used to test the established model. For this test, the wear values of the tools and the changes in spindle current were recorded. The average wear values of the four tool edges were taken as the wear data for each set. The raw spindle current data collected during machining are shown in Figure 13.

**Figure 14:**
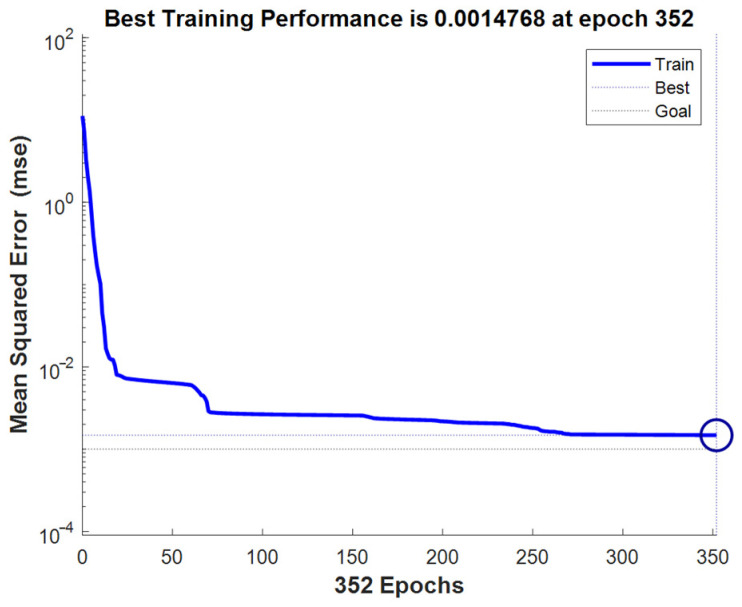
Training curves.

The experimental data recorded in Table 2 were used as the input for the VMD-BP neural network model established in Section 2.2. For model optimization and calibration, multiple iterations of learning and calibration were performed using known historical machining data and simulation data from the tool wear model. After the Mean Squared Error (MSE) was reduced to below 0.0015, tool condition predictions were made. The training curves are shown in Figure 14.

The flank wear width, VB, was used as input to predict the spindle power corresponding to the tool state under the given machining parameters. The predicted values are listed in Table 2.

**Figure 15:**
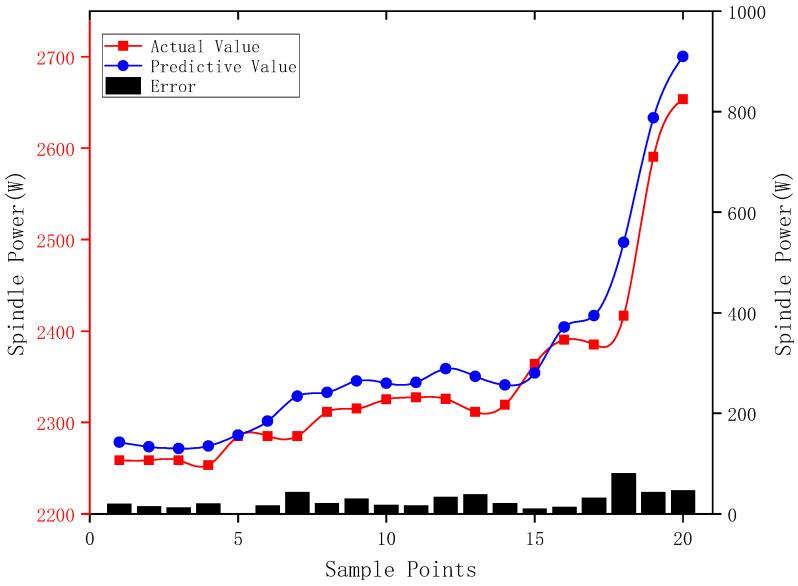
VMD-BP neural network model prediction graph.

From the prediction graph in Figure 15, it can be seen that the power prediction curve obtained from the model aligns closely with the actual power curve collected during machining. A comparison between the calculated data and the actual data shows an average error of 1.1256%, indicating the model’s excellent performance. Therefore, this model can be effectively applied to monitor tool wear conditions.

The table compares the experimental measurements of tool wear and power with the predicted values, illustrating the high accuracy of the VMD-BP model.

Before the research presented in this paper, the LSTM, ResNet, and combined ResNet-LSTM models were studied for prediction [31]. Although all three models achieve excellent prediction accuracy, they lack computational efficiency. To address this, the VMD-BP neural network model proposed in this paper ensures high prediction accuracy while significantly improving computational speed, making it suitable for guiding actual machining processes. As shown in Table 3, the VMD-BP neural network model demonstrates a significant advantage in terms of inference time.

## 5. Conclusions

This paper proposes a VMD-BP neural network prediction model based on spindle power data. The model uses spindle current data collected by a Hall current sensor to estimate spindle power, which is then input into the VMD-BP neural network model to predict real-time power during the machining process. The main conclusions are as follows:

(1) A VMD-BP neural network model was established, achieving high-accuracy real-time power prediction through training and calibration using historical and experimental data.

(2) The VMD method was employed to decompose the signals, enabling more accurate feature extraction. The most relevant features were selected as inputs to the prediction model, improving its accuracy.

(3) The model integrates advantages such as fast training speed, quick prediction speed, and high accuracy. The training phase takes approximately 1.5 h, while prediction takes about 0.15 s. This makes the system suitable for real-time tool wear monitoring in machining operations.

(4) The average error of the model is 1.1256%, confirming its reliability in practical applications. The output signal is the spindle power signal, allowing for direct observation and comparison of results. When the predicted power exceeds a predefined threshold, the system provides feedback to prompt machine shutdown for inspection, preventing excessive tool wear and reducing downtime.

Future work will focus on establishing a quantitative relationship between spindle power, tool wear, and machining surface quality, as well as exploring dynamic monitoring of surface roughness using spindle power data.

## Figures and Tables

**Table 1:** Process parameters for machining sample parts with tools.

Number	Spindle Speed (r/min)	Feed (mm/min)	Cutting Depth (mm)	Cutting Width (mm)
1	2000	400	0.2	6
2	2000	800	0.3	6
3	2000	600	0.4	6
4	3000	800	0.2	6
5	3000	600	0.3	6
6	3000	400	0.4	6
7	4000	600	0.2	6
8	4000	400	0.3	6
9	4000	800	0.4	6

**Table 2:** Comparison of measured data and predicted data.

Number	Tool Flank Wear VB (mm)	Spindle Power P (W)	Predicted Power P (W)
1	0.0275	2258.765	2278.495
2	0.0400	2258.765	2273.514
3	0.0475	2258.765	2271.529
4	0.0550	2253.500	2274.216
5	0.0653	2285.091	2286.321
6	0.0783	2285.091	2301.523
7	0.1018	2285.091	2328.795
8	0.1053	2311.418	2332.834
9	0.1162	2315.091	2345.375
10	0.1140	2325.091	2342.849
11	0.1149	2327.391	2343.882
12	0.1280	2325.622	2358.890
13	0.1206	2311.418	2350.422
14	0.1123	2319.418	2340.894
15	0.1237	2364.070	2353.973
16	0.1650	2390.397	2404.280
17	0.1750	2385.132	2416.959
18	0.2075	2416.723	2496.890
19	0.2563	2590.397	2633.235
20	0.2800	2653.620	2700.111
Average error	-	1.1256%	

**Table 3:** Comparison of tool wear prediction results.

Model	RMSE (mm)	*R* ^2^	Inference Time (s)
LSTM	0.0281	0.8744	0.5–0.9
Resnet	0.0182	0.9745	0.6–1.2
ResNet-LSTM	0.0101	0.9825	0.8–1.2
VMD-BP	0.0873	0.9190	0.1–0.2

## Data Availability

Data are contained within the article.
